# Cyclophosphamide pharmacokinetics and pharmacogenetics in children with B-cell non-Hodgkin's lymphoma

**DOI:** 10.1016/j.ejca.2015.12.007

**Published:** 2016-03

**Authors:** Gareth J. Veal, Michael Cole, Girish Chinnaswamy, Julieann Sludden, David Jamieson, Julie Errington, Ghada Malik, Christopher R. Hill, Thomas Chamberlain, Alan V. Boddy

**Affiliations:** a Northern Institute for Cancer Research, Newcastle University, Newcastle upon Tyne, United Kingdom; b Institute of Health and Society, Newcastle University, Newcastle upon Tyne, United Kingdom; c Tata Memorial Hospital, Mumbai, India; d Faculty of Pharmacy, The University of Sydney, NSW 2006, Australia

**Keywords:** Cyclophosphamide, B-cell NHL, Chemotherapy, Paediatrics, Pharmacokinetics, Pharmacogenetics

## Abstract

**Introduction:**

Variation in cyclophosphamide pharmacokinetics and metabolism has been highlighted as a factor that may impact on clinical outcome in various tumour types. The current study in children with B-cell non-Hodgkin's lymphoma (NHL) was designed to corroborate previous findings in a large prospective study incorporating genotype for common polymorphisms known to influence cyclophosphamide pharmacology.

**Methods:**

A total of 644 plasma samples collected over a 5 year period, from 49 B-cell NHL patients ≤18 years receiving cyclophosphamide (250 mg/m^2^), were used to characterise a population pharmacokinetic model. Polymorphisms in genes including *CYP2B6* and *CYP2C19* were analysed.

**Results:**

A two-compartment model provided the best fit of the population analysis. The mean cyclophosphamide clearance value following dose 1 was significantly lower than following dose 5 (1.83 ± 1.07 versus 3.68 ± 1.43 L/h/m^2^, respectively; mean ± standard deviation from empirical Bayes estimates; *P* < 0.001). The presence of at least one CYP2B6*6 variant allele was associated with a lower cyclophosphamide clearance following both dose 1 (1.54 ± 0.11 L/h/m^2^ versus 2.20 ± 0.31 L/h/m^2^, *P* = 0.033) and dose 5 (3.12 ± 0.17 L/h/m^2^ versus 4.35 ± 0.37 L/h/m^2^, *P* = 0.0028), as compared to homozygous wild-type patients. No pharmacokinetic parameters investigated were shown to have a significant influence on progression free survival.

**Conclusion:**

The results do not support previous findings of a link between cyclophosphamide pharmacokinetics or metabolism and disease recurrence in childhood B-cell NHL. While *CYP2B6* genotype was shown to influence pharmacokinetics, there was no clear impact on clinical outcome.

## Highlights

• The influence of cyclophosphamide clinical pharmacology on childhood cancer outcome has been investigated
• The presence of at least one CYP2B6*6 variant allele was associated with a lower rate of cyclophosphamide clearance
• Pharmacokinetic parameters investigated were not shown to have a marked influence on clinical outcome
• Findings do not support a link between cyclophosphamide metabolism and disease recurrence in B-cell non-Hodgkin's lymphoma

## 1 Introduction

The oxazaphosphorine alkylating agent cyclophosphamide is used across a wide range of tumour types in childhood cancer [1], [2]. In order for cyclophosphamide to exert its antitumour activity, the prodrug requires metabolic activation by hepatic cytochrome P-450 (CYP) enzymes to generate active alkylating species [3], [4]. Key enzymes involved in the initial metabolic step to form 4-hydroxycyclophosphamide, include CYP2B6, CYP2C19 and CYP3A4, expression of which can vary markedly between individuals [5]. The active metabolite 4-hydroxycyclophosphamide exists in equilibrium with its tautomeric form, aldophosphamide, with these metabolites transported to tumour cells via the systemic circulation [6]. Further spontaneous breakdown is required to form the active DNA-crosslinking metabolite, phosphoramide mustard, with the release of the urotoxic metabolite, acrolein [7]. A number of inactivating metabolic pathways may also play a role in determining cyclophosphamide efficacy, leading to the formation of 4-ketocyclophosphamide (KetoCP), dechloroethylcyclophosphamide (DCCP) and carboxyphosphamide (CXCP) [8]. The overall metabolism of cyclophosphamide is therefore a complex process, involving numerous enzymes which may vary in expression and activity in cancer patients.

Variation in the metabolism of cyclophosphamide between individuals has been highlighted as a factor that may impact on clinical outcome, in terms of both response and toxicity, in tumour types including breast cancer in adults and non-Hodgkin's lymphoma (NHL) in children. A study carried out in the United Kingdom (UK) by Yule *et al.*, previously indicated that lower clearance of cyclophosphamide to its active metabolites was associated with an increased risk of disease recurrence in paediatric B-cell NHL patients. In addition, likelihood of disease recurrence was positively related to a higher formation of inactive metabolites following cyclophosphamide administration [9].

A number of enzymes involved in the metabolism of cyclophosphamide, including CYPs, UGT and GST enzymes, may exhibit variable expression and activity between patients which could impact on cyclophosphamide metabolism and/or clinical response and toxicity [10]. Of particular note, *CYP2B6* and *CYP2C19* genotype have previously been shown to influence cyclophosphamide pharmacokinetics and activation, in terms of half-life of the parent drug, in breast cancer patients [11], [12].

The current study in children with B-cell NHL was designed to corroborate the findings of Yule *et al.* [9], in a larger prospective study incorporating genotyping for the common polymorphisms in genes known to influence the pharmacology of cyclophosphamide. While high cure rates are commonly seen in B-cell NHL with the use of cyclophosphamide-containing regimens [13], it is important to investigate factors that influence response rates and incidence of toxicity to further improve outcome in childhood cancer.

## 2 Patients and methods

### 2.1 Patient eligibility and treatment

Patients 18 years or younger, receiving cyclophosphamide as part of their standard clinical treatment for B-cell NHL, were eligible to participate in the trial. The study was approved by the UK Trent Multicentre Research Ethics Committee and registered through the appropriate clinical trial registries (PK 2005 02 – REC 04/MRE04/68; CTA: 17136/0243/001; EUDRACT: 2004-003731-31) prior to patient recruitment. Participating centres obtained written informed consent, either from patients or parents as appropriate, for all patients entered onto the study. Patients were required to have central venous access, in the form of double lumen central venous catheters, in order to participate in this pharmacokinetic study. Baseline toxicity data prior to cyclophosphamide treatment, including baseline haemoglobin, white blood cell and platelet counts, were obtained from patients' notes and details of concomitant medications prior to and/or in combination with cyclophosphamide were recorded. Additional patient characteristics and clinical parameters including glomerular filtration rate, serum creatinine, alanine transaminase (ALT) and bilirubin measurements were also collected for post-study analysis.

Cyclophosphamide (250 mg/m^2^) was administered as a 15 min infusion twice daily on days 2, 3 and 4 of treatment (six doses in total) as part of the COPADM regimen. This consisted of cyclophosphamide (1.5 g/m^2^ total dose as described, with hydration continued at 3000 ml/m^2^/day until 12 h after the final dose), vincristine (2.0 mg/m^2^; bolus intravenous infusion on day 1), prednisolone (60 mg/m^2^/day, days 1–7), doxorubicin (60 mg/m^2^, 1–6 h infusion on day 2), high dose methotrexate (3 g/m^2^, intravenous infusion over 3 h on day 1), folinic acid (15 mg/m^2^ orally every 6 h, beginning at 24 h from the start of methotrexate as required) and intrathecal methotrexate/hydrocortisone (8–15 mg on days 2 and 6). Toxicity following cyclophosphamide treatment was assessed by the National Cancer Institute Common Toxicity Criteria version 2.0. Progression free survival data were obtained from 6 monthly follow-up visits to the centre where treatment was undertaken.

### 2.2 Blood sampling and analysis

Blood samples (2.5 ml) for pharmacokinetic analysis were obtained from a central line prior to administration of the first dose of cyclophosphamide on day 2, at the end of infusion and at 1, 2, 4, 6 and 12 h after the start of infusion. Additional samples were obtained prior to administration of the first dose of cyclophosphamide on day 4 of treatment (dose 5), at the end of infusion and at 1, 2, 4, 6 and 12 h after the start of infusion of dose 5. All samples were taken from a different lumen from that used for drug administration following a standardised procedure. Plasma was separated from whole blood samples by centrifugation (1200*g*, 4 °C, 10 min) and stored at −20 °C prior to analysis. Samples were sent by overnight courier, on dry ice and in an insulated container, to the Northern Institute for Cancer Research, Newcastle University.

Concentrations of cyclophosphamide and its stable inactivated metabolites, KetoCP, DCCP and CXCP were measured in plasma using a validated LC/MS method as previously described [14]. Cyclophosphamide was obtained from Sigma (Poole, Dorset). The inactive metabolites and the internal standard deuterated cyclophosphamide (D_4_CP) were obtained from IIT (University of Bielefeld, Germany). The assay had a limit of quantification of 0.5 μg/ml for cyclophosphamide and 0.05 μg/ml for the metabolites and exhibited within- and between-run coefficients of variation and bias below 15%. QC samples for each analyte were included in each assay. Standard curves were linear between 0.5–10 μg/ml for cyclophosphamide and 0.05–1 μg/ml for CXCP, DCCP and KetoCP with r^2^ values ≥ 0.99. Samples containing concentrations of cyclophosphamide or metabolites above the linear range were diluted with blank plasma.

### 2.3 Pharmacokinetic analysis

A population pharmacokinetic model for cyclophosphamide was developed using nonlinear mixed effects modelling (NONMEM version 7.2), based on the enhancement of a model previously published by our group [14]. The first order conditional estimation method with η/ɛ interaction was used, together with ADVAN1/TRANS2 or ADVAN3/TRANS4 as appropriate. A composite error model was most appropriate to describe within-subject error. An additive error model, on the logarithmic scale, was used for inter-individual variability in pharmacokinetic parameters.

As cyclophosphamide concentrations were available from days 2 and 4 (doses 1 and 5) for each patient, additional error terms were included to account for inter-occasion variation (IOV) on clearance (CL) and volume of distribution in the central compartment (V1). The exact timing of the second dose administered on day 2 and of the doses administered on day 3 were not recorded. Given this unknown dosing history, to allow for non-zero cyclophosphamide concentrations prior to the start of the first cyclophosphamide infusion on day 4, a rate-controlled steady-state ‘infusion’ into the central compartment was assumed (terminating at the start of the current infusion). This has the effect of initialising all compartments with an appropriate amount of drug. The rate of the ‘infusion’ was allowed to vary across the population [15].

Allometric scaling was used for all population pharmacokinetic parameters; the approach taken to this scaling was the same as that used in a previously published analysis [16]. Changes in NONMEM objective function value (OFV), and examination of residual plots guided model structure development. Empirical Bayes estimates of pharmacokinetic parameters including CL, V_1_ and cyclophosphamide area under the plasma concentration-time curve (AUC) were obtained from the final population model. The covariates body weight, age, gender, ALT, bilirubin and creatinine, alongside genetic variation including CYP2B6*6 genotype were assessed for their relationship with CL.

Plasma concentrations of the inactive metabolites CXCP, DCCP and KetoCP were determined on day 2 (dose 1) and day 4 (dose 5) of cyclophosphamide treatment. Calculation of metabolite AUCs from time 0–6 h was carried out using the trapezoidal rule on day 2 (dose 1) and day 4 (dose 5).

### 2.4 Pharmacogenetics

Genomic DNA was obtained from whole blood samples using Qiagen QIAamp^®^ DNA Blood Maxi kits according to the manufacturer's instructions. DNA purity and concentration were measured using a NanoDrop ND-1000 (Thermo Scientific, Rockford, United States of America [USA]) and stored at −20 °C prior to pharmacogenetic analysis. Genotyping for SNPs CYP2B6*5 1459C > T (rs3211371), CYP2B6*6 785A > G (rs2279343) & 516G > A (rs3745274), CYP2C19*2 681G > A (rs4244285), CYP2C19*17-806C > T (rs12248560), GSTP1*2 313A > G (rs1695), *CAR* 540C > T (rs2307424) and *PXR*-25385C > T (rs3814055) were performed using TaqMan^®^ probes and an ABI 7500 Fast Real-Time PCR System (Applied Biosystems, California, USA) according to manufacturer's instructions. Allelic discrimination was performed using sequence detection software (Applied Biosystems).

### 2.5 Statistical analysis

For the analysis of pharmacogenetic data, overall differences between groups were assessed with the Mann–Whitney and Kruskal–Wallis tests using GraphPad Prism version 5.0 software (GraphPad Software, Inc., San Diego, CA, USA). Analysis of linkage disequilibrium was performed using Fisher's exact test (two-sided) for general contingency tables with SPSS version 15.0 software (SPSS Inc., Chicago, IL, USA). Time to disease progression was calculated as the delay between the first day of cyclophosphamide treatment and the first observation of disease progression or death. Hazard ratios for disease progression were estimated for individual covariates using the univariate Cox proportional hazards regression model approach, with each model fitted separately. Potential prognostic factors were tested as continuous variables. Statistical significance was given for P values < 0.05.

## 3 Results

### 3.1 Patient characteristics and treatment

A total of 49 patients receiving cyclophosphamide as part of their standard clinical treatment for B-cell NHL were entered onto the study over a 5 year period. Patients were recruited from nine UK centres. The study population had a median age of 11.7 years (range 3.5–18.7) and included 42 male and seven female patients. Patient characteristics including ethnicity, age, sex and body weight are provided in Table 1.

### 3.2 Pharmacokinetics

A population pharmacokinetic analysis using data collected from 48 patients on day 2 (dose 1) of cyclophosphamide treatment and 46 patients on day 4 (dose 5) of treatment was performed using NONMEM. Pharmacokinetic data were available following dose 1 and dose 5 from 45 patients, with a total of 644 plasma samples available for analysis.

The best fit of the population analysis was obtained with a two compartment model including random effects on CL, V_1_, Q and V_2_ allowing for correlation between CL and V1. The model included IOV for CL and V_1_. Allometric scaling was used to allow for differences in body size; population parameters are therefore scaled to a standard body surface area of 1.4 m^2^. Mean (coefficient of variation) parameters were CL 2.2 (37%) L/h/1.4 m^2^; V_1_ 18.4 (25%) L/1.4 m^2^; Q 5.2 (69%) L/h/1.4 m^2^ and V_2_ 21.8 (19%) L/1.4 m^2^. Estimates of IOV were CL 18% and V_1_ 21%. Estimates of the composite intra-subject error model were 0.1 μg/ml and 14% for the additive and multiplicative components respectively. There was a significant increase in CL on day 4 (OFV change of 116); on average CL increased by a factor of 2.1. No change was apparent for V_1_. The covariates body weight, age, gender, ALT and bilirubin were not observed to have a significant effect on cyclophosphamide pharmacokinetics. However, homozygous wild-type CYP2B6 patients were shown to have on average a 34% higher cyclophosphamide CL than patients with at least one variant allele (OFV change 7.7). Creatinine also had a relationship to CL (OFV change 10.6); as creatinine increased, CL tended to decrease.

Table 2 provides a summary of empirical Bayes estimates of cyclophosphamide pharmacokinetic parameters obtained from the final population model following dose 1 and dose 5. The pharmacokinetic parameter estimates obtained were in general agreement with values previously reported in the literature in paediatric patient populations. Cyclophosphamide Cmax values were observed between 15 min and 120 min following dose 1, ranging from 9.8–114.6 μg/ml and between 15 min and 60 min following dose 5, ranging from 8.53–53.7 μg/ml. The mean cyclophosphamide CL following dose 5 was significantly greater than that following dose 1 (3.68 ± 1.43 versus 1.83 ± 1.07 L/h/m^2^, respectively; *p* < 0.001). This shift in cyclophosphamide CL between doses 1 and 5 resulted in a significantly lower cyclophosphamide AUC following dose 5 (10.3 ± 5.3 mg/ml.min for dose 1 versus 4.7 ± 1.7 mg/ml.min for dose 5, p < 0.001). The volume of distribution in the central compartment (V_1_) was comparable following doses 1 and 5, with values of 17.3 ± 8.2 and 17.4 ± 7.9 observed, respectively.

Cyclophosphamide metabolites CXCP, DCCP and KetoCP could be quantified throughout the defined pharmacokinetic sampling period in all patients studied. A summary of cyclophosphamide metabolite AUC_0–6h_ values observed is provided in Table 3, with marked inter-patient variability observed for all metabolites. Significantly greater AUC_0–6h_ values were seen following dose 5 of cyclophosphamide treatment, as compared to dose 1, for all metabolites measured (CXCP – 198.9 ± 137.9 μg/ml.min for dose 5 versus 103.7 ± 60.9 μg/ml min for dose 1, *p* < 0.001; DCCP – 105.6 ± 60.9 μg/ml min versus 76.7 ± 49.6 μg/ml min, *p* = 0.008; KetoCP – 153.4 ± 61.3 μg/ml min versus 63.6 ± 27.5 μg/ml min, *p* < 0.001).

### 3.3 Pharmacogenetics

**Fig. 1:**
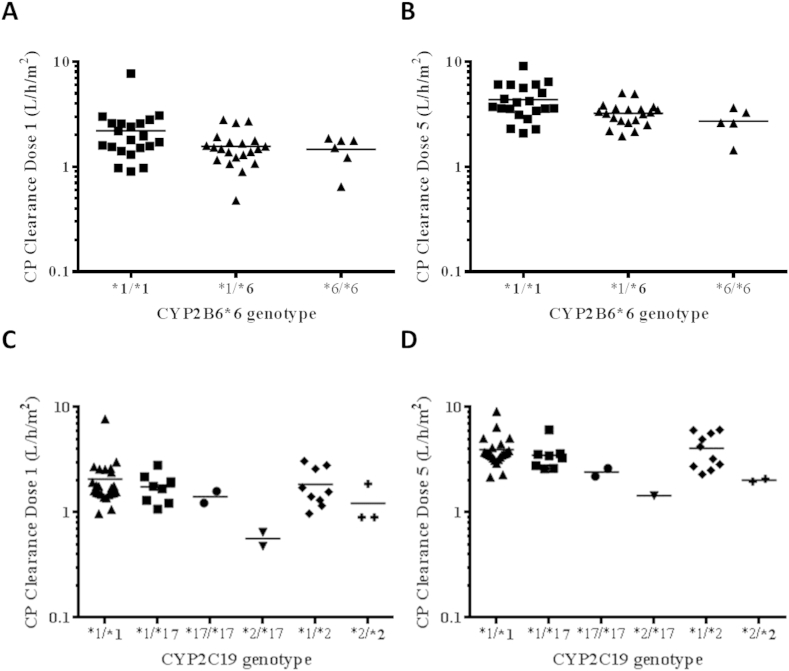
Effect of CYP2B6*6 genotype on cyclophosphamide clearance following dose 1 (A) and dose 5 (B) of treatment, and CYP2C19*2 and CYP2C19*17 genotype on cyclophosphamide clearance following dose 1 (C) and dose 5 (D) of treatment. The presence of at least one variant CYP2B6*6 allele was associated with a lower cyclophosphamide CL following both dose 1 (p = 0.033; panel A) and dose 5 (p = 0.0028; panel B), as compared to homozygous wild-type *CYP2B6* patients. No statistically significant influence on cyclophosphamide CL was found for CYP2C19*17 or CYP2C19*2 (panels C and D). The CYP2C19*2/*17 group (panels C and D) contains patients who are both *1/*2 and *1/*17.

The impact of pharmacogenetic variation on cyclophosphamide pharmacokinetics was investigated in all 49 patients studied, for whom both pharmacogenetic and pharmacokinetic data were available. Seven variant alleles of putative relevance for cyclophosphamide disposition were analysed. The allele frequencies for CYP2B6*5, CYP2B6*6, CYP2C19*2, CYP2C19*17, GSTP1*2, *CAR* 540C > T and *PXR*-25385C > T were in accordance with those observed previously in Caucasian populations and were consistent with Hardy-Weinberg equilibrium. For the 49 patients studied, the number of individuals with each individual CYP genotype studied were as follows: *1/*1 (40), *1/*5 (7) and *5/*5 (2) for CYP2B6*5; *1/*1 (21), *1/*6 (22) and *6/*6 (6) for CYP2B6*6; *1/*1 (34), *1/*2 (12) and *2/*2 (3) for CYP2C19*2 and *1/*1 (37), *1/*17 (10) and *17/*17 (2) for CYP2C19*17. Supplemental Table A.1 shows the individual genotype data and associated cyclophosphamide CL values for all patients studied. Relationships between cyclophosphamide CL following doses 1 and 5 of treatment and CXCP, DCCP and KetoCP AUC values and the studied genetic variants were investigated. A significant effect of CYP2B6*6 on cyclophosphamide CL was observed. The presence of at least one variant allele was associated with a lower cyclophosphamide CL following both dose 1 (1.54 ± 0.11 L/h/m^2^ versus 2.20 ± 0.31 L/h/m^2^, *p* = 0.033) and dose 5 (3.12 ± 0.17 L/h/m^2^ versus 4.35 ± 0.37 L/h/m^2^, *p* = 0.0028) of treatment, as compared to homozygous wild-type *CYP2B6* patients. It should be noted that within the CYP2B6*1/*1 patient group, there are a number of patients who are carriers of other *CYP2B6* variant alleles and similarly, within the CYP2B6*6 heterozygote group there are several patients who may be carriers of the CYP2B6*7 variant allele (see Supplemental Table A.1 for individual patient genotype data). We have not attempted to further stratify the patients into additional CYP2B6 groups based on *CYP2B6* genotype due to the limited number of patients involved. No other statistically significant influence on cyclophosphamide CL was found for any of the genetic variants investigated, including the previously reported effect of CYP2C19*17, although there was a trend towards a lower cyclophosphamide CL in patients with at least one CYP2C19*17 variant allele following both dose 1 (1.49 ± 0.19 L/h/m^2^ versus 1.94 ± 0.19 L/h/m^2^, *p* = 0.22) and dose 5 (3.12 ± 0.36 L/h/m^2^ versus 3.86 ± 0.25 L/h/m^2^, *p* = 0.13), as compared to homozygous wild-type *CYP2C19* patients. Nor was there any effect of genetic variation on CXCP, DCCP or KetoCP AUC values observed following doses 1 or 5 of treatment. Relationships between genotype for CYP2B6*6, CYP2C19*17 and CYP2C19*2 and cyclophosphamide CL following doses 1 and 5 of treatment are shown in Fig. 1.

### 3.4 Clinical response

**Fig. 2:**
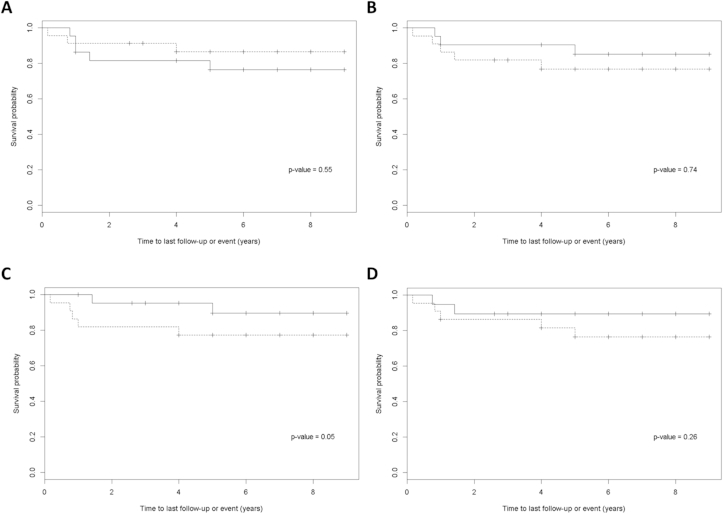
Kaplan–Meier curves of progression free survival according to (A) cyclophosphamide clearance following dose 1, (B) cyclophosphamide clearance following dose 5, (C) DCCP AUC_0–6h_ following dose 1 and (D) DCCP AUC_0–6h_ following dose 5. Patients with values above and below the median are displayed as dashed lines and solid lines, respectively. DCCP, dechloroethylcyclophosphamide; AUC, area under the plasma concentration-time curve.

Of the patients studied, 38/49 (78%) were alive with no disease at follow-up. The median time of follow-up was 6 years, with a range of 1–9 years. Of the remaining patients, two (4%) were alive with disease progression and seven (14%) had died following disease relapse or due to treatment-related deaths. The remaining two patients were lost to follow-up. In the Cox proportional hazards regression model, cyclophosphamide CL values following doses 1 or 5 had no significant influence on progression free survival. Cyclophosphamide metabolite AUC_0–6h_ values for patients alive with no disease and those who relapsed are listed in Table 3. DCCP AUC_0–6h_ determined following dose 1 had a negative prognostic effect on progression free survival (*p* = 0.05). However, this result should be treated with caution, bearing in mind the borderline significance and the fact that a total of eight variables were investigated. DCCP AUC_0–6h_ determined following dose 5 had no prognostic effect on progression free survival (*p* = 0.26). Fig. 2 shows Kaplan–Meier curves of progression free survival according to (A) cyclophosphamide CL following dose 1, (B) cyclophosphamide CL following dose 5, (C) DCCP AUC_0–6h_ following dose 1 and (D) DCCP AUC_0–6h_ following dose 5 of treatment, with patients stratified above and below the median values for each parameter. No other pharmacokinetic parameters investigated were shown to influence progression free survival. Similarly, there was no effect of genetic variation on clinical outcome for any of the genetic variants studied.

Mean DCCP AUC_0–6h_ values following doses 1 and 5 were 74% and 38% higher, respectively, in patients who relapsed as compared to patients who were alive with no disease at long-term follow-up (dose 1 − 122.0 ± 92.2 μg/ml min versus 70.1 ± 37.6 μg/ml min; dose 5 − 138.7 ± 86.8 μg/ml min versus 100.4 ± 55.6 μg/ml min). However, due to the level of inter-patient variability exhibited and the small number of patients in the relapsed group, these differences were not statistically significant.

## 4 Discussion

The metabolic elimination of cyclophosphamide has previously been identified as a factor which may influence exposure of both tumour and host tissues to active species and thus affect clinical outcome [5], [8], [9], [10], [11], [12]. Many of these studies have been in adult cancer patients across a number of different tumour types. One study in patients with childhood B-cell NHL found that cyclophosphamide CL and the extent of formation of inactive metabolites influenced the likelihood of disease relapse [9]. The current study was designed to corroborate these results in a larger prospective study, and to incorporate an investigation of genetic variants thought to influence the pharmacology of cyclophosphamide. This allowed us to address the hypothesis that pharmacogenetic variability, mediated by an effect on cyclophosphamide metabolism and pharmacokinetics, impacts on response to treatment.

A total of 49 children with B-cell NHL were recruited at nine UK centres over a 5 year period, with combined information generated on cyclophosphamide pharmacokinetics, metabolism, pharmacogenetics and clinical outcome. The length of time taken to complete the current study highlights the challenges faced when carrying out clinical pharmacology studies in a paediatric oncology setting, requiring the collection of serial blood samples from patients with a particular tumour type, receiving specific chemotherapeutics on a defined treatment protocol. The main aim of the study was to investigate the direct influence of pharmacogenetics on the pharmacokinetics and metabolism of cyclophosphamide, based on existing pharmacokinetic and clinical response data in a comparable patient population. In contrast to the Yule study, no correlation was observed between cyclophosphamide clearance following either dose 1 or dose 5 of treatment and patient outcome, with no difference in clearance observed in patients alive and disease-free at long-term follow-up, as compared to patients who had experienced disease relapse. While cyclophosphamide pharmacokinetics were shown to be influenced by CYP2B6*6 genotype, with the presence of at least one variant allele associated with a lower cyclophosphamide CL, the variations in cyclophosphamide CL observed had no apparent bearing on clinical response. No other effect of pharmacogenetic variation on cyclophosphamide pharmacokinetics or metabolite formation was observed. Previous studies have reported modest effects of both *CYP2B6* and *CYP2C19* genotype on the pharmacokinetics of anticancer drugs, including cyclophosphamide, with the impact being both dose dependent and potentially influenced by disease status [5], [11], [12], [17], [18], [19].

The extent of cyclophosphamide metabolism in the current study was comparable to data from previous publications in children, with significant increases in metabolite formation observed over several days of treatment due to the induction of CYP enzymes [14], [20]. Although DCCP AUC_0–6h_ following dose 1 of treatment was associated with worse progression free survival, the level of significance and number of tests carried out warn against drawing firm conclusions from this result. Similarly, although DCCP AUC_0–6h_ values following doses 1 and 5 of treatment were higher in patients who relapsed as compared to patients who were alive with no disease at long-term follow-up, these differences were not statistically significant. No other relationships between the extent of cyclophosphamide metabolism and clinical outcome were observed. Again, these data contrast with the previous findings of Yule *et al.* in a comparable patient population [9]. Although a more sensitive LCMS assay was used in the current study, with a ten-fold lower limit of quantification for cyclophosphamide metabolites, this should not have influenced the ability to detect an underlying difference in metabolite levels between relapse and disease-free patients.

Other disparities between the current study and the Yule *et al*. publication include differences in cyclophosphamide dose and patient gender and a high percentage of Burkitt's lymphoma patients in the current study. A lower dose of cyclophosphamide was administered to patients in the current study (250 mg/m^2^ versus 1000 mg/m^2^) and a lower percentage of females were included as compared to the previous study (14% versus 42%, respectively). Cyclophosphamide dose and gender have previously been reported to influence both pharmacokinetics and metabolism of the drug and therefore may explain the differences in findings between the two studies [20], [21]. In particular, the association between cyclophosphamide pharmacokinetics and clinical outcome in breast cancer patients was in the setting of high-dose chemotherapy (6000 mg/m^2^) [22]. Based on the potential for saturation of metabolism at an increased dose of cyclophosphamide, it is certainly possible that the more frequent administration of lower drug doses in the current study may explain our findings. Beyond these factors the patient populations were comparable in terms of age ranges and other patient characteristics. In terms of data analysis the current study included the more robust use of a univariate Cox proportional hazards regression model to assess the impact of cyclophosphamide pharmacokinetic parameters and metabolite levels on clinical outcome, as opposed to the direct comparison of median values between relapse and remission patients in the previous study.

The current study involved quantification of cyclophosphamide alongside its inactive metabolites, predominantly in an attempt to replicate the data generated by Yule *et al.* [9] and recognising the difficulty of accurately quantifying the unstable metabolites in a multi-centre study. While other investigators have suggested that CXCP (also known as CEPM) may be a marker of cyclophosphamide bioactivation [23], [24], [25], in the current study there was no link between CXCP plasma concentrations and clinical outcome. Interestingly, there were proportionally larger increases observed in CXCP and ketoCP AUC_0–6h_ values between cyclophosphamide dose 1 and dose 5, as compared to the increase in DCCP AUC_0–6h_. Cyclophosphamide auto-induction models have previously been studied in some detail by Huitema *et al.* [26], [27].

In summary, the findings of the current study do not support a link between cyclophosphamide metabolism and recurrence of disease in childhood B-cell NHL with the cyclophosphamide dosing regimen described. While the influence of *CYP2B6* genotype on cyclophosphamide pharmacokinetics was confirmed, there was no clear impact of pharmacogenetic variation on clinical outcome.

## Role of the funding source

This work was supported in part by Cancer Research UK, the North of England Children's Cancer Research Fund and the Experimental Cancer Medicine Centre Network. No funding bodies played a role in the study design, the collection, analysis or interpretation of data, the writing of the report or the decision to submit the article for publication.

## Conflict of interest statement

The authors have no financial relationships relevant to the work contained in this article to disclose and no other conflicts of interest to disclose.

## Figures and Tables

**Table 1:** Patient characteristics.

Characteristic	No. of patients	%
Evaluable patients	49	
Age (years)		
3–8	13	26.5
8–12	13	26.5
11+	23	47
Sex		
Male	42	86
Female	7	14
Ethnicity		
White British	42	86
White other	2	4
Asian Indian	1	2
Asian Pakistani	1	2
Mixed background	3	6
BW (kg)		
Median	39.2	
Range	16.3–138
BSA (m^2^)		
Median	1.25	
Range	0.69–2.2
Diagnosis		
Burkitt's lymphoma	42	86
Diffuse large B-cell lymphoma	7	14

BW – body weight; BSA – body surface area.

**Table 2:** Cyclophosphamide pharmacokinetic parameters. Summary statistics for empirical Bayes estimates obtained from final population pharmacokinetic model.

Study dose	Age (years)	BW (kg)	BSA (m^2^)	Dose (mg)	CL (L/h/m^2^)	V_1_ (L)	AUC (mg/ml min)
1	11.2 ± 4.0	40.8 ± 22.5	1.24 ± 0.38	314 ± 95	1.83 ± 1.07	17.3 ± 8.2	10.3 ± 5.3
5	11.3 ± 4.0	41.1 ± 21.9	1.25 ± 0.37	314 ± 92	3.68 ± 1.43	17.4 ± 7.9	4.7 ± 1.7

BW – body weight; BSA – body surface area; CL – clearance; V_1_ – volume of distribution in the central compartment; AUC – area under the plasma concentration-time curve; SD – standard deviation. All values indicate mean ± SD.

**Table 3:** Cyclophosphamide metabolite formation following doses 1 and 5 of treatment in study patients alive with no disease as compared to relapsed patients.

		Dose 1 AUC_0–6h_ (μg/ml.min)			Dose 5 AUC_0–6h_ (μg/ml.min)		
CXCP	DCCP	KetoCP	CXCP	DCCP	KetoCP
All patients (n = 49)	Mean	103.7	76.7	63.6	198.9	105.6	153.4
SD	60.9	49.6	27.5	137.9	60.9	61.3
Range	35.4–352	21.4–285	19.9–141	90.6–921	37.5–287	69.8–353
Alive no disease (n = 38)	Mean	103.9	70.1^a^	62.2	199.0	100.4	148.1
SD	63.2	37.6	26.4	144.4	55.6	58.9
Relapsed (n = 9)	Mean	102.4	122.0^a^	71.5	198.1	138.7	182.5
SD	47.1	92.2	34.5	97.5	86.8	70.9

CXCP – carboxyphosphamide; DCCP – dechloroethylcyclophosphamide; KetoCP – ketocyclophosphamide; SD – standard deviation; AUC – area under the plasma concentration-time curve.

a DCCP AUC_0–6h_ determined following dose 1 had a negative prognostic effect on progression free survival (*p* = 0.05).
