# Supplementary material for: Cyclophosphamide pharmacokinetics and pharmacogenetics in children with B-cell non-Hodgkin's lymphoma
Source: Eur J Cancer. 2016 Mar;55:56–64. doi: 10.1016/j.ejca.2015.12.007 (PMC4778608; doi:10.1016/j.ejca.2015.12.007)
Supplement: Supplementary file 1 [file mmc1.docx]

**Supplemental Table A.1. Compiled genotype and cyclophosphamide clearance data listed by SNP**

| **Study ID** | **CPM**  **Dose 1 CL (L/hr/m^2^)** | **CPM**  **Dose 5 CL (L/hr/m^2^)** | **CYP2B6 *4** | **CYP2B6**  ***9** | **CYP2B6**  ***6** | **CYP2B6**  ***5** | **CYP2C19**  ***2** | **CYP2C19**  ***17** | **CAR 540C>T** | **GSTP1*2** | **PXR -25385C>T** |
| --- | --- | --- | --- | --- | --- | --- | --- | --- | --- | --- | --- |
| 1 | 0.648 | 1.442 | *4/*4 | *9/*9 | *6/*6 | *1/*1 | *1/*2 | *1/*17 | CT | GG | TT |
| 2 | 2.187 | 3.610 | *1/*1 | *1/*1 | *1/*1 | *1/*1 | *1/*1 | *1/*17 | TT | AA | CT |
| 3 | 1.766 | 3.654 | *4/*4 | *9/*9 | *6/*6 | *1/*1 | *1/*1 | *1/*1 | CT | AA | CT |
| 4 | 1.516 | 3.124 | *1/*1 | *1/*1 | *1/*1 | *1/*1 | *1/*1 | *1/*1 | TT | AA | CC |
| 5 | 0.896 | 1.958 | *1/*4 | *1/*9 | *1/*6 | *1/*1 | *2/*2 | *1/*1 | CT | AA | CC |
| 6 | 1.474 | 3.346 | *1/*4 | *1/*9 | *1/*6 | *1/*1 | *1/*1 | *1/*1 | CC | AA | CT |
| 7 | 1.594 | 3.416 | *1/*1 | *1/*1 | *1/*1 | *1/*1 | *1/*1 | *1/*1 | CT | AA | CC |
| 8 | 1.586 | 2.202 | *1/*4 | *1/*9 | *1/*6 | *1/*1 | *1/*1 | *17/*17 | TT | GA | CT |
| 9 | 1.225 | 2.622 | *4/*4 | *9/*9 | *6/*6 | *1/*1 | *1/*1 | *1/*17 | TT | AA | CC |
| 10 | 1.388 | 3.509 | *1/*4 | *1/*9 | *1/*6 | *1/*1 | *1/*1 | *1/*1 | TT | GA | TT |
| 11 | 0.899 | 2.085 | *1/*1 | *1/*1 | *1/*1 | *5/*5 | *2/*2 | *1/*1 | CT | GA | CT |
| 12 | 1.528 | 2.920 | *1/*4 | *1/*9 | *1/*6 | *1/*1 | *1/*1 | *1/*1 | CT | GA | CC |
| 13 | 3.013 | 6.445 | *1/*1 | *1/*1 | *1/*1 | *1/*1 | *1/*1 | *1/*1 | TT | GG | CC |
| 14 | 2.614 | 5.050 | *1/*4 | *1/*9 | *1/*6 | *1/*1 | *1/*1 | *1/*1 | TT | GA | CC |
| 15 | ND | 2.503 | *1/*4 | *1/*9 | *1/*6 | *1/*1 | *1/*2 | *1/*1 | CC | GA | CT |
| 16 | 1.379 | ND | *1/*4 | *1/*9 | *1/*6 | *1/*1 | *1/*1 | *1/*1 | CT | GG | CT |
| 17 | 0.973 | 2.275 | *1/*1 | *1/*1 | *1/*1 | *1/*1 | *1/*1 | *1/*1 | CT | AA | CC |
| 18 | 2.799 | 4.941 | *1/*4 | *1/*9 | *1/*6 | *1/*1 | *1/*2 | *1/*1 | CT | GA | CT |
| 19 | 1.567 | 3.575 | *1/*1 | *1/*1 | *1/*1 | *1/*1 | *1/*1 | *1/*1 | CT | GA | CT |
| 20 | 2.600 | 5.634 | *1/*1 | *1/*1 | *1/*1 | *1/*5 | *1/*2 | *1/*1 | TT | AA | CT |
| 21 | 0.970 | 2.292 | *1/*1 | *1/*1 | *1/*1 | *1/*1 | *1/*2 | *1/*1 | CT | AG | TT |
| 22 | 0.477 | ND | *1/*4 | *1/*9 | *1/*6 | *5/*5 | *1/*2 | *1/*17 | CT | GG | CT |
| 23 | 1.519 | 3.278 | *4/*4 | *9/*9 | *6/*6 | *1/*1 | *1/*1 | *1/*1 | TT | AG | CC |
| 24 | 1.308 | 2.860 | *1/*1 | *1/*1 | *1/*1 | *1/*5 | *1/*2 | *1/*1 | CT | AG | CT |
| 25 | 1.074 | 3.291 | *1/*4 | *1/*9 | *1/*6 | *1/*1 | *1/*1 | *1/*17 | CT | AG | CC |
| 26 | 7.723 | 9.087 | *1/*1 | *1/*1 | *1/*1 | *1/*1 | *1/*1 | *1/*1 | CT | AG | CT |
| 27 | 1.713 | 6.077 | *1/*1 | *1/*1 | *1/*1 | *1/*1 | *1/*2 | *1/*1 | CT | AA | CC |
| 28 | 1.667 | 3.455 | *1/*4 | *1/*9 | *1/*6 | *1/*1 | *1/*1 | *1/*17 | CT | AA | CT |
| 29 | 1.866 | ND | *4/*4 | *9/*9 | *6/*6 | *1/*1 | *2/*2 | *1/*1 | TT | AA | TT |
| 30 | 1.548 | 3.605 | *1/*1 | *1/*1 | *1/*1 | *1/*5 | *1/*1 | *1/*1 | TT | AA | CC |
| 31 | 1.157 | 2.743 | *1/*4 | *1/*9 | *1/*6 | *1/*1 | *1/*2 | *1/*1 | TT | GG | CC |
| 32 | 1.778 | 3.704 | *1/*4 | *1/*9 | *1/*6 | *1/*1 | *1/*1 | *1/*1 | TT | GG | CC |
| 33 | 1.303 | 2.785 | *1/*4 | *1/*9 | *1/*6 | *1/*5 | *1/*1 | *1/*17 | CT | AA | CT |
| 34 | 1.481 | 3.591 | *1/*4 | *1/*9 | *1/*6 | *1/*1 | *1/*1 | *1/*1 | CT | AA | CT |
| 35 | 2.404 | 4.114 | *1/*1 | *1/*1 | *1/*1 | *1/*1 | *1/*1 | *1/*1 | CT | AG | CC |
| 36 | 2.563 | 3.721 | *1/*1 | *1/*1 | *1/*1 | *1/*1 | *1/*1 | *1/*1 | CC | AG | CC |
| 37 | 2.706 | 3.167 | *1/*4 | *1/*9 | *1/*6 | *1/*5 | *1/*1 | *1/*1 | TT | AA | CT |
| 38 | 1.565 | 3.219 | *1/*4 | *1/*9 | *1/*6 | *1/*1 | *1/*2 | *1/*1 | CT | GG | TT |
| 39 | 1.227 | 2.623 | *1/*4 | *1/*9 | *1/*6 | *1/*1 | *1/*1 | *17/*17 | CT | AG | TT |
| 40 | 1.798 | 4.440 | *1/*1 | *1/*1 | *1/*1 | *1/*1 | *1/*1 | *1/*1 | CT | AA | CT |
| 41 | 1.772 | 2.598 | *4/*4 | *9/*9 | *6/*6 | *1/*1 | *1/*1 | *1/*17 | TT | AG | TT |
| 42 | 2.576 | 5.054 | *1/*1 | *1/*1 | *1/*1 | *1/*1 | *1/*1 | *1/*1 | TT | AA | TT |
| 43 | 1.065 | 2.158 | *1/*4 | *1/*9 | *1/*6 | *1/*5 | *1/*1 | *1/*1 | CT | AG | CT |
| 44 | 1.705 | 3.854 | *1/*4 | *1/*9 | *1/*6 | *1/*1 | *1/*1 | *1/*1 | TT | AA | CC |
| 45 | 1.408 | 4.230 | *1/*1 | *1/*1 | *1/*1 | *1/*1 | *1/*2 | *1/*1 | CT | AG | CC |
| 46 | 1.953 | 3.557 | *1/*1 | *1/*1 | *1/*1 | *1/*5 | *1/*1 | *1/*17 | CT | AA | CT |
| 47 | 3.070 | 6.030 | *1/*1 | *1/*1 | *1/*1 | *1/*1 | *1/*2 | *1/*1 | CT | AA | CC |
| 48 | 1.921 | 3.498 | *1/*4 | *1/*9 | *1/*6 | *1/*1 | *1/*1 | *1/*1 | CT | AA | CC |
| 49 | 2.818 | 6.088 | *1/*1 | *1/*1 | *1/*1 | *1/*1 | *1/*1 | *1/*17 | CT | AG | CT |
